# Editorial: MicroRNAs as New Players in Endocrinology

**DOI:** 10.3389/fendo.2018.00459

**Published:** 2018-08-17

**Authors:** Chun Peng, Yan-Ling Wang

**Affiliations:** ^1^Department of Biology and Centre for Research in Biomolecular Interactions, York University, Toronto, ON, Canada; ^2^State Key Laboratory of Stem Cell and Reproductive Biology, Institute of Zoology, Chinese Academy of Sciences, Beijing, China

**Keywords:** microRNA, cell signaling, endocrine system, hormone-like activity, miRceptor

Endocrinology is the study of hormones. The term “hormone” was coined by Starling in 1905, based on the discovery of secretin (1). Hormones are traditionally defined as chemical messengers produced by endocrine glands (ductless glands) and enter circulation to regulate the activity of target organs at a distal site. However, not all hormones are produced from classical endocrine glands, and many chemical messengers exert their effects locally via autocrine/paracrine regulation. Therefore, endocrinology has become a science that deals more broadly with cell-cell communications and cellular signaling mechanisms (1). Hormones usually exert their functions by interacting with receptors located on the membrane or inside the cells to initiate signaling cascades, leading to changes in cellular activity (1).

MicroRNAs (miRNAs) are non-coding RNAs that play critical roles in gene expression (2, 3). In animal cells, microRNAs primarily regulate gene expression by binding to miRNA response elements, usually found at the 3′ UTR of target mRNAs, to decrease mRNA stability and induce translational repression (4, 5). It is now clear that miRNAs are crucial regulators of many developmental events and physiological processes. Several lines of evidence support the critical roles of miRNAs in the endocrine system.

First, miRNAs regulate hormone production, activity, and target cell responsiveness. miRNAs can directly target genes encoding hormones or enzymes involved in hormone production or metabolism, thereby affecting hormone concentrations. Proteins that modulate hormone actions, such as antagonists, can also be regulated by miRNAs. Furthermore, miRNAs target hormone receptors and intracellular signaling molecules to alter target cell responses. For example, we have shown that Nodal, a member of the transforming growth factor-β (TGF-β) superfamily, is targeted by miR-378a-5p (6). On the other hand, Nodal modulator1 (NOMO1), which is an antagonist of Nodal signaling, is a target of miR-675 (7). We have also identified several miRNAs that target Nodal receptors (8–10). Other members of the TGF-β family, as well as many other signaling pathways have also been reported to be regulated by miRNAs (11–17).

Second, many studies have demonstrated that miRNAs are regulated by hormones (18–20), and such regulation can occur at the level of transcription (21) or processing (22). For example, Interleukin 6 activates STAT3 to induce the transcription of miR-21 and miR-181-b1 genes (21). SMAD, which is activated by TGF-β signaling, regulates DROSHA-mediated miRNA maturation (22). Interestingly, the hormones-regulated miRNAs can be part of the feedback pathway to fine tune hormone activity. Thyroid hormone (TH) represses the transcription of miR-21, which in turn, downregulates the expression of *GRHL3*, a transcriptional inhibitor of type 3 iodothyronine deiodinase (D3). D3 inactivates TH, thereby terminating TH action (23).

Finally, miRNAs have been shown to have hormone-like activities and are important for intercellular communication. They can be secreted by host cells into extracellular fluids where they are either transported by vesicles, such as microvesicles and exosomes, or by forming complexes with proteins, especially AGO2 (24). These extracellular miRNAs can be taken up by recipient cells to regulate their activities. For example, it has been demonstrated that miRNAs produced from cardiac stem cells can be transferred, via gap junctions, to nearby cells and regulate target gene expression in the recipient cells (25). Thus, miRNAs may have endocrine, paracrine, and autocrine regulatory functions, similar to hormones (26). Interestingly, some miRNAs have been reported to bind to Toll-like receptors (TLR) and induce downstream signaling pathways (27). However, it remains to be determined if other miRceptors exist and what determines the specificity of miRNA-target cell interaction.

The Research Topic highlights some of the recent findings about miRNAs and their relationships with the endocrine system. O'Brien et al. provide an updated overview of miRNA biogenesis and mode of actions, as well as their circulation in the extracellular fluid. Park discusses the mechanisms of miRNA secretion via exocytosis and the functions of extracellular miRNAs in cellular signaling. Yusof et al. provide an example of miRNA expression in normal and cancerous thyroid gland. Tang et al. report findings from a primary study that elucidates how estrogen protects cardiomyocytes from oxidative stress via miR-494. Liu et al. present results that demonstrate miR-518b regulates MAPK signaling. Another study by Peng et al. shows that Interferon γ (IFNγ) induces miR-146a, which then regulates NFκB signaling. Neale et al. highlight the role of ghrelin in critical limb ischmia and the involvement of miRNAs in this process. Finally, Toms et al. summarize the interplay between ovarian miRNAs and steroid hormones in regulating the cyclic activity of the ovary.

## Author contributions

All authors listed have made a substantial, direct and intellectual contribution to the work, and approved it for publication.

### Conflict of interest statement

The authors declare that the research was conducted in the absence of any commercial or financial relationships that could be construed as a potential conflict of interest.
